# Individualised prescription of medications for treatment of obesity in adults

**DOI:** 10.1007/s11154-023-09808-2

**Published:** 2023-05-18

**Authors:** Samantha Hocking, Priya Sumithran

**Affiliations:** 1 Faculty of Medicine and Health, University of Sydney, Camperdown, New South Wales Australia; 2 Department of Endocrinology, Royal Prince Alfred Hospital, Camperdown, New South Wales Australia; 3 Department of Medicine, (St Vincent’s Hospital), University of Melbourne, VIC Fitzroy, Australia; 4 Department of Endocrinology, Austin Health, Heidelberg, Victoria Australia; 5 Department of Surgery, Central Clinical School, Monash University, Level 6, 99 Commercial Rd, Melbourne, Victoria 3004 Australia

**Keywords:** Obesity medications, Weight loss, Pharmacotherapy, Precision medicine

## Abstract

Obesity continues to increase in prevalence globally, driven by changes in environmental factors which have accelerated the development of obesity in individuals with an underlying predisposition to weight gain. The adverse health effects and increased risk for chronic disease associated with obesity are ameliorated by weight loss, with greater benefits from larger amounts of weight reduction. Obesity is a heterogeneous condition, with the drivers, phenotype and complications differing substantially between individuals. This raises the question of whether treatments for obesity, specifically pharmacotherapy, can be targeted based on individual characteristics. This review examines the rationale and the clinical data evaluating this strategy in adults. Individualised prescribing of obesity medication has been successful in rare cases of monogenic obesity where medications have been developed to target dysfunctions in leptin/melanocortin signalling pathways but has been unsuccessful in polygenic obesity due to a lack of understanding of how the gene variants associated with body mass index affect phenotype. At present, the only factor consistently associated with longer-term efficacy of obesity pharmacotherapy is early weight loss outcome, which cannot inform choice of therapy at the time of medication initiation. The concept of matching a therapy for obesity to the characteristics of the individual is appealing but as yet unproven in randomised clinical trials. With increasing technology allowing deeper phenotyping of individuals, increased sophistication in the analysis of big data and the emergence of new treatments, it is possible that precision medicine for obesity will eventuate. For now, a personalised approach that takes into account the person’s context, preferences, comorbidities and contraindications is recommended.

## Introduction

Obesity affects more than 650 million people worldwide [1]. Its prevalence is estimated to have nearly tripled since 1975 [1] and it is predicted that by 2030, one in five women and one in seven men globally – equating to more than 1 billion people - will be living with obesity [2].

Obesity is widely recognized as a chronic disease, as well as being a risk factor for many other conditions, such as type 2 diabetes mellitus (T2D), non-alcoholic fatty liver disease (NAFLD, including hepatic steatosis, steatohepatitis and cirrhosis), cardiovascular disease, hypertension, osteoarthritis, obstructive sleep apnoea, cholelithiasis and several types of cancer [3]. Around one-fifth of preventable deaths and disability-adjusted life years from chronic diseases are attributed to excess weight [2].

Fortunately, treatment of obesity reduces many of these complications. Improvements in health and quality of life are seen with as little as 3-5% weight loss, and are generally progressive with greater weight loss of up to 25% [4, 5]. A range of treatment modalities is recommended in clinical practice guidelines for obesity management, including lifestyle interventions, medications and bariatric surgery, to achieve and maintain weight loss [6, 7]. Therapeutic goals and strategy are primarily based on the degree and complications of excess weight, comorbidities and contraindications, and patient preferences. Financial considerations, such as insurance coverage and out-of-pocket costs, must also be taken into account.

It is well-recognised that individual responses to all treatment modalities are heterogeneous. An increasing number of treatment options and advances in technology have led to growing interest in personalising treatment for obesity to optimise benefits and safety. Here, we will review the rationale for individualised prescription of obesity medications, and the clinical data evaluating this approach in adults.

## Search strategy

Papers for this review were identified from a search of Web of Science using the search terms: “individuali*”, “precision”, “pharmacogenomic”, “obesity pharmacotherapy”, “obesity medication” and approved obesity medications or combinations (orlistat, phentermine, bupropion, liraglutide, semaglutide). Peer-reviewed journal articles published in English before January 10, 2023 were reviewed and included on the basis of relevance to the review topic. Precision nutrition/lifestyle interventions, individualisation of bariatric surgical procedures, and interventions in children/adolescents are beyond the scope of this review.

## Heterogeneity of obesity

Obesity is defined as “abnormal or excessive accumulation of body fat that presents a risk to health” [1]. In practice, obesity is classified by body mass index (BMI), calculated as a person's weight in kilograms divided by the square of height in meters (kg/m^2^). BMI is a useful population-level measure of excess weight, as it correlates well with body fatness [8, 9] and is calculated the same way regardless of sex and age in adulthood. However, it has limitations, including that it may not correspond to the same degree of body fat in athletic compared with non-athletic individuals, people of different ethnicities will have varying amounts of body fat at the same BMI, and it gives no indication of the regional distribution of adipose tissue within the body. These factors are important because the excess accumulation of adipose tissue in visceral depots, and ectopic storage of fat in non-adipose organs including the liver, skeletal muscle, heart and pancreas, are more strongly linked to metabolic and cardiovascular disease than overall adiposity [10, 11]. For these reasons, obesity defined by BMI alone is a remarkably heterogeneous condition across individuals.

### Heterogeneity in body fat distribution and relationship to obesity complications.

Fat distribution varies with sex, pubertal development, race, age and disease states, and in response to drugs and hormones [12]. When compared to White Europeans of the same BMI, Asians have 3 – 5% higher body fat [13], and South Asians in particular are more prone to develop abdominal obesity [14]. In contrast, non-Hispanic Blacks have lower body fat and higher lean muscle mass than White Europeans at the same BMI [15]. For this reason, there is debate about whether ethnic specific cut-points to define overweight and obesity by BMI should be used to ensure that recommendations for surveillance and management of obesity and associated conditions are appropriate for patients in minority ethnic populations [13, 16].

Central obesity is associated with increased risk for cardiometabolic disease, cancers and mortality compared with peripheral obesity, irrespective of whether an individual has a healthy body weight, overweight or obesity [17, 18]. In the setting of excess energy intake, both subcutaneous and visceral fat stores expand. When the capacity for adipocyte recruitment and hypertrophy is overwhelmed, fat accumulates in ectopic sites such as visceral depots, the liver, skeletal muscle and pancreatic beta cells which is accompanied by inflammation, insulin resistance and other features of the metabolic syndrome [10]. Put simply, all fat is not the same.

A consequence of this is that obesity, as defined by BMI alone, includes a group of individuals with BMI>30 kg/m^2^ and an excess amount of body fat who are otherwise metabolically healthy. This ‘metabolically healthy obesity’ (MHO) has an estimated prevalence of 10 – 40% depending on the population studied and the definition used [19, 20]. MHO is characterised by a preservation of insulin sensitivity despite an increase in adiposity. It has been postulated that individuals with MHO are protected from the ‘spillover’ of excess energy stores into visceral and ectopic sites due to an increase in the capacity of their eutopic adipose tissue sites to store fat, therefore avoiding the adverse metabolic consequences of a higher fat mass [12, 21]. However, protection from metabolic disease does not ensure that individuals with MHO are immune to mechanical complications of obesity and psychological distress. Furthermore, it is unclear whether individuals with MHO followed longitudinally remain ‘healthy’ with increasing duration of obesity [22, 23].

### The genetics of obesity

Obesity is a heritable trait, with heritability estimates varying according to the population studied. In studies based on the comparison of pairs of monozygotic and dizygotic twins reared apart or together, the hereditability of obesity is estimated to be 50 – 90% [24]. In contrast, adoption studies produce the lowest estimates of heritability with a range of 10% to 35% and nuclear family studies yield intermediate, although overlapping estimates, ranging from 30 – 50%. A possible reason for this wide range in estimates is that heritability varies across classes of BMI [24]. This is suggested by evidence that the risk of obesity (>90^th^ centile of BMI distribution) is about 3 fold for individuals with a family history of obesity, and 5 – 8 fold for individuals with a family history of severe obesity (>95^th^ percentile) compared with individuals with family members of normal weight [25]. That obesity is more heritable in individuals with a higher BMI was confirmed by recent data from the Framingham cohort, which found the genetic heritabilities of anthropometric (BMI, waist-to-height ratio and waist-to-hip ratio) and imaging (computed tomography and dual-energy X-ray absorptiometry) measures of adiposity increase with increasing adiposity [26]. Therefore, the heritability of obesity is estimated at 30% to 35% for individuals with normal BMI, increasing to 50% in individuals with overweight, 60% to 65% for class I obesity and 80% for class II and III obesity [24].

**Fig. 1:**
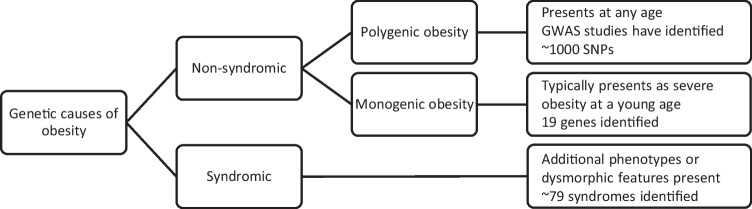
Schematic representation of genetic predisposition to obesity

In most people, the predisposition to obesity involves several genes (Fig. 1). Polygenic obesity (also known as common obesity) is the result of hundreds of polymorphisms that each have a small effect on body weight. To date, genome wide association studies have identified close to 1000 near-independent single nucleotide polymorphisms (SNPs) associated with BMI that together explain ∼6.0% of the variance of BMI [27]. For the vast majority of these loci, it remains unknown which genes are causal, what cells, tissues and organs they act in to affect body weight, and by what underlying mechanisms.

Rarely, obesity is caused by mutations in a single gene, so-called monogenic or Mendelian obesity. Monogenic obesity typically presents as severe obesity at a young age (<10 years) and is characterised by hyperphagia. Most (but not all) cases are due to deficiency in a gene of the leptin-melanocortin signaling pathway, a major player in the regulation of energy balance [28]. Thus far, defects in 19 genes have been identified in monogenic obesity [29], which is estimated to be responsible, in aggregate, for 5% to 10% of severe early-onset obesity cases in populations of European descent [24], with a higher predicted prevalence in populations with a high level of consanguinity. In contrast to polygenic obesity, knowledge of the causative gene in monogenetic obesity can precisely guide treatment (see later section on medications for monogenic obesity).

### Complex etiology of obesity

The fundamental cause of obesity and overweight is an imbalance between energy consumed and energy expended over a prolonged time period. However, the mechanisms that underlie this positive energy balance are complex, numerous, overlapping, and mostly beyond willful control. Genetic risk scores perform poorly in predicting an individual’s future risk of obesity, suggesting that environmental factors play a crucial role in the development of obesity [30]. Globally, there has been an increased intake of energy-dense, highly palatable, cheap, more-processed and effectively marketed foods that are high in fat and sugars. Some have proposed an increase in physical inactivity due to the increasingly sedentary nature of many forms of work, changing modes of transportation, and increasing urbanization [31]. Overweight and obesity are the expected outcome of these drivers in predisposed individuals.

## Overview of medications for obesity management

Medications for obesity management are indicated in conjunction with lifestyle intervention for adults with a BMI ≥30 kg/m^2^ or those with a BMI ≥27 kg/m^2^ and at least one complication of excess weight. Six agents are widely available for weight management in adults, some of which are also indicated for the treatment of obesity in adolescents in certain regions. These medications are reviewed in more detail elsewhere [32], and summarised in Table 1. In addition, on the basis of data from phase 3 clinical trials [33, 34], regulatory approval is expected to be sought in 2023 for an obesity indication for tirzepatide, a dual GLP-1/GIP agonist already approved in the U.S. for management of T2D.

**Table 1:** Currently available obesity medications [35−40]

**Medication**	**Orlistat** **(Xenical**^**®**^** 120 mg; Alli**^**®**^** 60 mg)**	**Phentermine** **(Duromine**^**®**^** Metermine**^**®**^**)**	**Phentermine-topiramate ER** **(Qsymia**^**®**^**)**	**Naltrexone-Bupropion (Contrave**^**®**^** Mysimba**^**®**^**)**	**Liraglutide 3 mg** **(Saxenda**^**®**^**)**	**Semaglutide 2.4 mg (Wegovy**^**®**^**)**
**Regulatory approval**	FDA, EMA, Australia Central and South America	FDA, Australia	FDA	FDA, EMA, Australia, Central and South America	FDA, EMA, Australia, Central and South America	FDA, EMA, Australia
**Dosage form and dosing**	60-120 mg three times a day, with meals, oral	15, 30, 37.5 (or 40) mg once daily, oral	Up to 15 mg phentermine–92 mg topiramate once daily, oral	16 mg naltrexone–180 mg bupropion twice a day, oral	3 mg, once daily, subcutaneous	2.4 mg once weekly, subcutaneous
**Mechanism of action**	Inactivation of gastric and pancreatic lipase	Stimulation of the release of noradrenaline, serotonin, and dopamine	Sympathomimetic and GABA receptor activation, carbonic anhydrase inhibition	Opioid receptor antagonist; dopamine and noradrenaline reuptake inhibitor	Central control of appetite and peripheral effects through GLP-1 receptor agonism in hypothalamus and hindbrain; also slows gastrointestinal transit and enhances glucose metabolism	

**Key indications**	BMI ≥30 kg/m^2^ or BMI ≥27 kg/m^2^ with at least one weight-related health condition					
	>12 years of age*	>16 years of age*	>12 years of age*	>18 years of age*	>12 years of age*	>12 years of age*
Average placebo-subtracted weight loss at ~12 months (%)*	4% at 1 year (120 mg tds) [30]	6% at 20 weeks [31]	9% at 1 year [32]	5% at 1 year [33]	6% at 1 year [34]	12.5% at 68 weeks [35]
Proportion of participants with 5% and 10% weight loss at ~12 months*	73 and 41% (vs 45 and 21% with placebo)	Not reported	67 and 47% (vs 17 and 7% with placebo)	48 and 25% (vs 16 and 7% with placebo)	63 and 33% (vs 27 and 11% with placebo)	86 and 69% (vs 31 and 12% with placebo)
**Contraindications**	Pregnancy, lactation	Coronary artery disease, uncontrolled hypertension, cardiac arrhythmias, hyperthyroidism, glaucoma, MAOI, not recommended with SSRI, pregnancy, lactation	As for phentermine, plus glaucoma, history of renal stones, hyperthyroidism and within 14 days of treatment with monoamine oxidase inhibitors, pregnancy, lactation	Uncontrolled hypertension, seizure disorders, bipolar disorder, undergoing abrupt discontinuation of alcohol or anti-convulsant drugs, chronic opioid use, MAOI, pregnancy, lactation	Personal or family history of medullary thyroid carcinoma or MEN 2, pregnancy, lactation	As for liraglutide
**Adverse effects**	Steatorrhea, oily spotting, faecal urgency, fat-soluble vitamin deficiency	Dry mouth, insomnia, palpitations, tachycardia, hypertension, anxiety, dizziness, small risk of primary pulmonary hypertension	As for phentermine, plus paraesthesia, dysgeusia (altered taste), memory loss, depression, fetal abnormalities	Nausea, constipation, headache, vomiting, dizziness, insomnia, dry mouth, diarrhoea, hypertension, seizures, precipitation of mania	Nausea, diarrhea, constipation, vomiting, headache, dyspepsia, abdominal pain	As for liraglutide

(modified from Perdomo et al. [32] Lancet 2023;401:1116-30)

*EMA* European Medicines Agency, *FDA* Food and Drug Administration, *GABA* gamma-aminobutyric acid, *MAOI* monoamine oxidase inhibitor, *MEN 2* Multiple endocrine neoplasia type 2, *SSRI* Selective serotonin reuptake inhibitors, *T2D* Type 2 diabetes

*indicates FDA age approval

The currently available agents facilitate modification of eating behaviour and weight loss via reduced energy absorption, reduced hunger, increased satiety and/or reduced rewarding properties of energy-dense food. All reduce weight and improve health, but they have differing mechanisms of action, leading to distinct profiles of both beneficial and adverse effects (Table 1).

Mean placebo-subtracted weight losses in clinical trials are ~18% for tirzepatide, 13% for semaglutide and 4-6% for the older agents, but there are very few studies comparing the available agents with one another [33, 35, 37−40]. The only two comparative randomised trials have found larger mean weight losses with liraglutide 3 mg daily compared with orlistat 120 mg tds (mean difference 3.7 kg at 1 year) [41] and semaglutide 2.4 mg weekly compared with liraglutide 3 mg daily (mean difference 8.5 kg at 68 weeks) [42]. Regardless of mean weight losses, treatment responses vary widely between individuals, along a normally distributed (bell-shaped) curve, with a considerable proportion of participants losing at least 10% of total body weight over 1 year of treatment for all medications (Table 1).

## Individualised prescription of obesity medications

The well-recognised heterogeneity of the contributors, manifestations and complications of obesity, and variability in individual responses to treatment, raise the question of whether management approaches can be developed to match the right obesity medication to the right individual. Development of an individualised management plan is a fundamental part of clinical practice. This is particularly important for multifactorial, complex, chronic conditions for which there is a range of therapeutic options, as is the case for obesity.

However, there is currently no reliable method of identifying which medication will be most effective for any given patient prior to initiating treatment. Early treatment response (weight loss at 16 weeks) is the only factor consistently associated with longer-term weight outcome [43, 44], hence regulatory authorities recommend stopping most obesity medications if less than 5% weight loss has been achieved after 12-16 weeks, but this does not assist with the initial choice of medication.

Treatment goals should always be individualised, but in general terms, the main goal of obesity management is usually to improve health and quality of life. These improvements are largely related to the amount of weight loss achieved. While some beneficial outcomes, such as prevention of type 2 diabetes in people with pre-diabetes and reduced triglycerides can occur with weight loss of 5% [4, 45], these parameters improve progressively with greater weight loss. Weight loss of 10% or more is generally required for substantial improvements in other complications of obesity, such as non-alcoholic steatohepatitis, obstructive sleep apnoea, joint pains due to osteoarthritis, and health-related quality of life [46]. Therefore, for patients with complications of obesity, treatments with the greatest efficacy in achieving and sustaining weight loss are likely to be the preferred initial treatment, unless there are contraindications or other reasons to choose against them.

Other clinically important considerations include patient phenotype (e.g. medical and psychosocial history, concurrent medications, and preferences) and medication characteristics (e.g. side effect profile, mode of administration). For example, a history of epilepsy or bipolar disorder are contraindications to use of naltrexone-bupropion; previous pancreatitis or a needle phobia make liraglutide, semaglutide and tirzepatide less suitable; uncontrolled hypertension excludes phentermine, phentermine-topiramate and naltrexone-bupropion.

Even for obesity medications with similar mean weight losses, the agents differ in their effects on cardiometabolic parameters, due to their diverse mechanisms of action. For example, GLP-1RAs stimulate insulin release from pancreatic beta cells, and are therefore associated with greater glycaemic improvements than other agents, hence they are likely to be the preferred class of medication for obesity management in people with pre-diabetes. Naltrexone-bupropion is associated with less reduction in mean blood pressure for the same degree of weight loss as other agents [47]. As bupropion is used as an aid to smoking cessation, patients who are concurrently trying to quit smoking may be good candidates for naltrexone-bupropion. In practice, the choice of medication is often heavily influenced by financial constraints or limited to agents covered by insurance or government subsidies.

The idea of selecting medications to target the phenotypic characteristics most relevant to obesity in each individual, such as the main drivers of their eating behaviours (e.g. hunger, impaired satiety, food cravings, food reward), is appealing. However, as yet, there are no randomised studies examining whether this approach could improve treatment outcomes. Moreover, several medications appear to have overlapping beneficial actions on these factors, with reduction in food cravings and reward-related eating having been shown for naltrexone-bupropion, phentermine and semaglutide [38, 48, 49], although there are no head to head comparisons of these actions. Nonetheless, small, proof-of concept studies suggest that this goal has potential.

Among 12 patients treated with phentermine-topiramate for 2 weeks, energy intake at an *ad libitum* buffet meal prior to treatment was negatively correlated with weight loss at 2 weeks [50]. In a subsequent real-world observational study [51], participants (n=84) treated for obesity in a clinical service underwent baseline assessment of energy intake at an *ad libitum* buffet meal, affect (Hospital Anxiety and Depression Scale [HADS]), gastric emptying of a mixed meal and resting energy expenditure [REE, indirect calorimetry]). Participants were categorised into four phenotypic groups according to cut-offs derived from a larger (n=100) cohort and prescribed medication according to phenotype as follows: >75^th^ percentile of energy intake ‘hungry brain’ (phentermine-topiramate or lorcaserin) or HADS score ‘emotional hunger’ (naltrexone-bupropion); <25^th^ percentile of gastric emptying ‘hungry gut’ (liraglutide) or REE ‘slow burn’ (phentermine plus resistance exercise). Treatment outcomes were compared against a group of patients (n=228) who received standard care (obesity medication choice based on standard criteria, e.g. side effect profile, glycaemia, patient preference). At 12 months of follow-up, mean weight loss was 15.9% in the phenotype-guided group compared with 9.0% in the standard care cohort.

These results await confirmation in randomised clinical trials. Advances in technology will allow continued gains in the ability to perform detailed phenotypic characterisation, which may potentially enable even more precise tailoring of the treatment to the person, to maximise effectiveness and minimise adverse effects.

### Individualised prescription for monogenic obesity

The most successful example to date of a precision medicine approach to obesity treatment comes from monogenic obesity syndromes.

Recombinant human leptin is transformative for individuals with leptin deficiency due to mutations in the leptin gene. In these exceedingly rare patients, leptin treatment results in reductions in hyperphagia, body weight and fat mass, and restoration of endocrine function [52].

Setmelanotide, a selective agonist of the melanocortin 4 receptor (MC4R), acts as a substitute for the absent melanocyte-stimulating hormone (MSH) in patients with pro-opiomelanocortin (POMC) deficiency owing to loss-of-function mutations in the POMC or PCSK1 genes, and in patients with mutations in the leptin receptor (LEPR), which is essential for POMC function. In clinical trials, treatment with setmelanotide results in substantial reduction in hunger and weight loss of ≥10% after 1 year of treatment in 80% of individuals with POMC deficiency and 45% of those with LEPR deficiency [53]. It has been estimated that in the USA, >12,800 individuals carry loss-of-function mutations in the melanocortin pathway for whom setmelanotide may be more effective for weight loss than any other treatment [54].

In contrast, for polygenic (common) obesity, most SNPs have not been linked with clinical endpoints and therefore their discovery has yet to translate into similar clinical breakthroughs, or to guiding the choice of treatment.

### Pharmacogenomics

Natural variation in genes can influence the effects of a particular treatment in an individual. Pharmacogenomics refers to “the study of variations of DNA and RNA characteristics as related to drug response” [55]. Drug response includes pharmacokinetic profile as well as beneficial and adverse effects. Studying associations between variants in genes for drug-metabolising enzymes, transporters and receptors, and medication responses, can inform the choice and dose of medications, to improve the safety (and potentially the efficacy) of drug treatments.

Pharmacogenomic markers are already included in drug labelling for >500 medications (predominantly in the field of oncology [56], and are used in clinical practice for specific conditions. For example, people with variants in the dihydropyrimidine dehydrogenase gene that result in deficiency of this enzyme are prone to developing severe adverse reactions to fluoropyrimidine-based chemotherapies such as 5-fluorouracil, and capecitabine which are metabolised by the enzyme DPD. In some countries, routine pre-treatment testing is recommended for patients undergoing treatment for cancers with these agents [57].

For obesity medications, there are a few studies indicating that gene variants may be associated with treatment responses. Variants of the GLP-1 receptor gene have been associated with weight loss response and gastric emptying in people treated with liraglutide [58-60]. A variant in the insulin receptor gene (INSR) was associated with weight loss in clinical trials of topiramate for obesity management [61].

Although sibutramine has been withdrawn from the market, pharmacogenomic studies have identified several gene variants associated with weight loss response. In a Taiwanese population, variants in the adiponectin and uncoupling protein 2 genes have been associated with body fat loss [62, 63]. Four studies have shown an association between weight loss response to sibutramine and the C825T variant of the GNB3 gene (which is associated with increased activation of the G-protein β3 subunit [64-67]). However, findings regarding the direction of this association have not been consistent, with a greater weight loss response to sibutramine in CC genotype carriers in one study [64], greater weight and fat loss in TC and TT genotypes in two studies [65, 66], and greater weight loss but less fat mass loss in TT and TC genotypes in another [67]. These differences may relate to different durations of treatment and different ethnicities of the populations studied, and highlight that translating pharmacogenomic data into treatment recommendations will not be straightforward.

## Can precision approaches to type 2 diabetes inform the treatment of obesity?

As obesity and T2D are complex diseases that share genetic and environmental risks, progress in precision prescribing of diabetes therapies may reflect the future of obesity medication prescription. Similar to obesity, the greatest advances in precision medicine in diabetes care are in monogenic forms of diabetes in which targeted therapies are particularly effective [68]. However, for the majority of individuals with T2D, variants in many genes contributing to numerous metabolic pathways contribute to the risk of developing T2D, with each genetic variant exerting modest effects on clinical outcomes. Therefore, it is not surprising that findings from genome-wide association studies (GWAS) have thus far not translated to clinical guidance on optimal choice of therapy for T2D.

Another approach has been to cluster individuals into subtypes of diabetes based on phenotypic characteristics at diagnosis. This approach has identified five subtypes of T2D: an autoimmune form (covering type 1 diabetes and other related clinical entities), two severe forms (one dominated by insulin deficiency, the other by insulin resistance), and two milder forms (termed “obesity-related” and “age-related” diabetes) [69]. The utility of the clustering approach to predict response to therapy has been explored using clinical trial data from the ADOPT and RECORD studies. Clusters differed in glycaemic response, with a particular benefit for thiazolidinediones in patients in the severe insulin-resistant diabetes cluster and for sulfonylureas in patients in the mild age-related diabetes cluster. However, using simple clinical features outperformed assigning an individual to a subgroup to select therapy with models combining four simple clinical measures (age, sex, baseline glycated haemoglobin [HbA1c], and BMI) explaining more variation in response than did the clusters [70].

The MASTERMIND consortium has demonstrated the utility of clinical variables in predicting response to therapy with sulfonylureas, thiazolidinediones, SGLT-2 inhibitors and DPP-IV inhibitors [71, 72]. A limitation of these studies is that a single clinical variable (e.g. HbA1c) is used as the measure of response, which does not take into account the benefits of choice of treatment on other cardiometabolic risk factors or diabetes complications. Furthermore, such approaches neglect other important considerations (such as cost, side effects, patient preference, or comorbidities) when choosing treatment, which in some cases may be regarded as equally, if not more important, than the selected clinical variable. That said, these studies suggest that similar methodology could be applied utilising data from previously conducted obesity trials to identify clinical features predictive of medication response. This will require consideration of the outcomes of most relevance to the goals of treatment, which are broader than weight loss itself (i.e. improvements in health and quality of life, which are partly but not entirely related to amount of weight loss).

## Perspective: precision medicine as a goal for obesity treatment

At present, our ability to predict an individual’s response to any given medication prior to treatment initiation is insufficient to inform the choice of agent. Rapid advances in technology are likely to provide clinical tools that enable more widespread in-depth phenotyping of people with obesity. Standardising these tools and outcome measures to enable pooling of datasets [73, 74], and increased sophistication in the analysis of big data, will undoubtedly aid in identifying more consistent predictors of response. Moreover, in coming years, we will have a wider range of effective medications for obesity management with different mechanisms of action [75] that could potentially be matched to an individual’s phenotype. However, whether this greater complexity and precision will translate to specific treatment strategies and better health outcomes (particularly outcomes of importance to patients and clinicians) compared with the current approach to medication selection is uncertain. Furthermore, the development of a new generation of obesity medications which result in >10-15% weight loss (and associated health improvements) in the majority of people who are treated challenges the need to identify individuals most likely to respond. This goal was arguably more important when only a minority of patients achieved clinically meaningful weight loss in response to obesity pharmacotherapy.

Most precision medicine initiatives aimed at identifying genetic, epigenetic, metabolomic, proteomic, microbiome (among others) profiles of people with obesity have predominantly considered White European populations, which constitute a minority of people with obesity globally. Not only will other populations need to be considered before such characteristics are translated into treatment strategies, we need to consider where the development of such strategies should sit among priorities for the treatment of polygenic (common) obesity. Although uncommon, screening for monogenic obesity should be considered for children who present with severe, early-onset obesity, particularly with a phenotype of hyperphagia, as targeted therapies are now available and result in improved outcomes.

Obesity is common and increasing in prevalence, most rapidly in low- and middle-income countries [2]. Even if precision at an individual level were feasible for a disease that affects nearly one in six of the world’s adults [2], consideration must be given to how precision approaches to obesity medicine will avoid exacerbating health inequities. At present, only a small minority of people can access effective treatment at all [2, 76]. Ensuring the provision of equitable access to effective, evidence-based medications will be expensive [77]. In the development and evaluation of new treatment models and strategies, scalable improvements in healthcare delivery are likely to be more cost-effective and have a greater beneficial impact on the health of the world’s population than precision approaches that can only be applied to a small number of individuals. Patient preferences, comorbidities, contraindications, and psychological, social and environmental context will always remain important considerations. Rather than precision medicine, a personalised approach that takes these factors into account may be more suited to the treatment of obesity.

## Abbreviations

BMI: body mass index
GWAS: genome-wide association studies
HbA1c: glycated haemoglobin
HADS: Hospital Anxiety and Depression Scale
MC4R: melanocortin 4 receptor
MHO: metabolically healthy obesity
MSH: melanocyte-stimulating hormone
NAFLD: non-alcoholic fatty liver disease
POMC: pro-opiomelanocortin
SNPs: single nucleotide polymorphisms
T2D: type 2 diabetes mellitus

## References

[1] Health Organization W. Obesity and overweight. 2023. Available from: https://www.who.int/news-room/fact-sheets/detail/obesity-and-overweight.

[2] Federation WO. World Obesity Atlas 2022. 2022. World Obesity Federation: London.

[3] Fruhbeck G (2013). Obesity: The Gateway to Ill Health - an EASO Position Statement on a Rising Public Health, Clinical and Scientific Challenge in Europe. Obes Facts.

[4] Wing RR (2011). Benefits of Modest Weight Loss in Improving Cardiovascular Risk Factors in Overweight and Obese Individuals With Type 2 Diabetes. Diabetes Care.

[5] Kolotkin RL (2001). The relationship between health-related quality of life and weight loss. Obes Res.

[6] Yumuk V (2015). European Guidelines for Obesity Management in Adults. Obes Facts.

[7] Wharton S (2020). Obesity in adults: a clinical practice guideline. Canadian Med Assoc J.

[8] Garrow JS, Webster J (1985). Quetelet index (w/h-2) as a measure of fatness. Int J Obes.

[9] Flegal KM, Graubard BI (2009). Estimates of excess deaths associated with body mass index and other anthropometric variables. Am J Clin Nutri.

[10] Longo M, et al. Adipose Tissue Dysfunction as Determinant of Obesity-Associated Metabolic Complications. Int J Mol Sci2019;20(9). 10.3390/ijms20092358 PMC6539070 31085992

[11] Fruhbeck G (2019). The ABCD of Obesity: An EASO Position Statement on a Diagnostic Term with Clinical and Scientific Implications. Obes Facts.

[12] Sakers A (2022). Adipose-tissue plasticity in health and disease. Cell.

[13] Barba C (2004). Appropriate body-mass index for Asian populations and its implications for policy and intervention strategies. Lancet.

[14] Raji A (2001). Body fat distribution and insulin resistance in healthy Asian Indians and Caucasians. J Clin Endocrinol Metab.

[15] Wagner DR, Heyward VH (2000). Measures of body composition in blacks and whites: a comparative review. Am J Clin Nutri.

[16] Caleyachetty R (2021). Ethnicity-specific BMI cutoffs for obesity based on type 2 diabetes risk in England: a population-based cohort study. Lancet Diabetes Endocrinol.

[17] Sahakyan KR, et al. Normal-Weight Central Obesity: Implications for Total and Cardiovascular Mortality. Ann Int Med. 2015;163(11):827-+. 10.7326/M14-2525 PMC4995595 26551006

[18] Barberio AM, et al. Central body fatness is a stronger predictor of cancer risk than overall body size. Nat Commun. 2019;10. 10.1038/s41467-018-08159-w PMC6342989 30670692

[19] Blundell JE (2014). Beyond BMI - Phenotyping the Obesities. Obes Facts.

[20] Bluher M (2020). Metabolically Healthy Obesity. Endoc Rev.

[21] Cypess AM (2022). Reassessing Human Adipose Tissue. New England J Med.

[22] Kramer CK, Zinman B, Retnakaran R. Are Metabolically Healthy Overweight and Obesity Benign Conditions? A Systematic Review and Meta-analysis. Ann Int Med. 2013;159(11):758-+. 10.7326/0003-4819-159-11-201312030-00008 24297192

[23] Chang Y (2012). Impact of BMI on the incidence of metabolic abnormalities in metabolically healthy men. Int J Obes.

[24] Bouchard C (2021). Genetics of Obesity: What We Have Learned Over Decades of Research. Obesity.

[25] Lee JH, Reed DR, Price RA (1997). Familial risk ratios for extreme obesity: implications for mapping human obesity genes. Int J Obes.

[26] Williams PT (2020). Quantile-dependent heritability of computed tomography, dual-energy x-ray absorptiometry, anthropometric, and bioelectrical measures of adiposity. Int J Obes.

[27] Yengo L (2018). Meta-analysis of genome-wide association studies for height and body mass index in similar to 700 000 individuals of European ancestry. Human Mol Genet.

[28] Loos RJF, Yeo GSH (2022). The genetics of obesity: from discovery to biology. Nat Rev Genet.

[29] Paolacci S, et al. Mendelian non-syndromic obesity. Acta Biomed 2019;90(10-S):87-89. 10.23750/abm.v90i10-S.8766 PMC7233639 31577261

[30] Loos RJF, Janssens A (2017). Predicting Polygenic Obesity Using Genetic Information. Cell Metab.

[31] Popkin BM, Ng SW. The nutrition transition to a stage of high obesity and noncommunicable disease prevalence dominated by ultra-processed foods is not inevitable. Obes Rev 2022;23(1). 10.1111/obr.13366 PMC8639733 34632692

[32] Perdomo CM (2023). Contemporary medical, device, and surgical therapies for obesity in adults. Lancet.

[33] Jastreboff AM, et al. Tirzepatide Once Weekly for the Treatment of Obesity. New England J Med. 10.1056/NEJMc2211120 36239655

[34] le Roux CW (2023). Tirzepatide for the treatment of obesity: Rationale and design of the SURMOUNT clinical development program. Obesity.

[35] Torgerson JS (2004). XENical in the prevention of diabetes in obese subjects (XENDOS) study. Diabetes Care.

[36] Weintraub M (1984). a double-blind clinical-trial in weight control - use of fenfluramine and phentermine alone and in combination. Arch Intern Med.

[37] Allison DB (2012). Controlled-Release Phentermine/Topiramate in Severely Obese Adults: A Randomized Controlled Trial (EQUIP). Obesity.

[38] Greenway FL (2010). Effect of naltrexone plus bupropion on weight loss in overweight and obese adults (COR-I): a multicentre, randomised, double-blind, placebo-controlled, phase 3 trial. Lancet.

[39] Pi-Sunyer X, et al. A Randomized, Controlled Trial of 3.0 mg of Liraglutide in Weight Management. New England J Med. 2015;373(1):11-22. 10.1056/NEJMoa1411892 26132939

[40] Wilding JPH (2021). Once-Weekly Semaglutide in Adults with Overweight or Obesity. New England J Med.

[41] Astrup A (2012). Safety, tolerability and sustained weight loss over 2 years with the once-daily human GLP-1 analog, liraglutide. Int J Obes.

[42] Rubino DM (2022). Effect of Weekly Subcutaneous Semaglutide vs Daily Liraglutide on Body Weight in Adults With Overweight or Obesity Without Diabetes The STEP 8 Randomized Clinical Trial. Jama-J Am Med Assoc.

[43] Fujioka K, et al. Early Weight Loss with Liraglutide 3.0 mg Predicts 1-Year Weight Loss and is Associated with Improvements in Clinical Markers. Obesity 2016;24(11):2278-2288. 10.1002/oby.21629 PMC5129670 27804269

[44] Fujioka K (2016). The relationship between early weight loss and weight loss at 1 year with naltrexone ER/bupropion ER combination therapy. Int J Obes.

[45] Hamman RF (2006). Effect of weight loss with lifestyle intervention on risk of diabetes. Diabetes Care.

[46] Ryan DH, Yockey SR (2017). Weight Loss and Improvement in Comorbidity: Differences at 5%, 10%, 15%, and Over. Current Obes Rep.

[47] Apovian CM (2013). A Randomized, Phase 3 Trial of Naltrexone SR/Bupropion SR on Weight and Obesity-related Risk Factors (COR-II). Obesity.

[48] Moldovan CP (2016). Effects of a Meal Replacement System Alone or in Combination with Phentermine on Weight Loss and Food Cravings. Obesity.

[49] Friedrichsen M, et al. The effect of semaglutide 2.4 mg once weekly on energy intake, appetite, control of eating, and gastric emptying in adults with obesity. Diabetes Obes Metab 2021;23(3):754-762. 10.1111/dom.14280 PMC7898914 33269530

[50] Acosta A, et al. Quantitative Gastrointestinal and Psychological Traits Associated With Obesity and Response to Weight-Loss Therapy. Gastroenterology. 2015. 148(3):537-+. 10.1053/j.gastro.2014.11.020 PMC4339485 25486131

[51] Acosta A (2021). Selection of Antiobesity Medications Based on Phenotypes Enhances Weight Loss: A Pragmatic Trial in an Obesity Clinic. Obesity.

[52] Farooqi IS (2002). Beneficial effects of leptin on obesity, T cell hyporesponsiveness, and neuroendocrine/metabolic dysfunction of human congenital leptin deficiency. J Clin Invest.

[53] Clement K (2020). Efficacy and safety of setmelanotide, an MC4R agonist, in individuals with severe obesity due to LEPR or POMC deficiency: single-arm, open-label, multicentre, phase 3 trials. Lancet Diabetes Endocrinol.

[54] Ayers KL (2018). Melanocortin 4 Receptor Pathway Dysfunction in Obesity: Patient Stratification Aimed at MC4R Agonist Treatment. J Clin Endocrinol Metab.

[55] Agency EM, ICH Topic E15. Definitions for genomic biomarkers, pharmacogenomics, pharmacogenetics, genomic data and sample coding categories. Eur Med Agency. 2007. London.

[56] Administration USFaD. Table of Pharmacogenomic Biomarkers in Drug Labeling. 2023. Available from: https://www.fda.gov/drugs/science-and-research-drugs/table-pharmacogenomic-biomarkers-drug-labeling.

[57] Agency EM, EMA recommendations on DPD testing prior to treatment with fluorouracil, capecitabine, tegafur and flucytosine. EMA/229267/2020. Euro Med Agency. 2020.

[58] Chedid V, et al. Allelic variant in the glucagon-like peptide 1 receptor gene associated with greater effect of liraglutide and exenatide on gastric emptying: A pilot pharmacogenetics study. Neurogastroenterol Motility 2018;30(7). 10.1111/nmo.13313 PMC6003833 29488276

[59] de Luis DA (2015). Evaluation of weight loss and metabolic changes in diabetic patients treated with liraglutide, effect of RS 6923761 gene variant of glucagon-like peptide 1 receptor. J Diabetes Its Complications.

[60] Jensterle M (2015). Genetic variability in GLP-1 receptor is associated with inter-individual differences in weight lowering potential of liraglutide in obese women with PCOS: a pilot study. Eur J Clin Pharmacol.

[61] Li QQS (2016). A candidate-gene association study of topiramate-induced weight loss in obese patients with and without type 2 diabetes mellitus. Pharmacogenet Genom.

[62] Hsiao TJ (2010). A common variant in the adiponectin gene on weight loss and body composition under sibutramine therapy in obesity. Clin Pharmacol-Adv App.

[63] Hsiao TJ (2010). Effect of the Common-866G/A Polymorphism of the Uncoupling Protein 2 Gene on Weight Loss and Body Composition under Sibutramine Therapy in an Obese Taiwanese Population. Mol DiagTher.

[64] Hauner H (2003). Prediction of successful weight reduction under sibutramine therapy through genotyping of the G-protein beta 3 subunit gene (GNB3) C825T polymorphism. Pharmacogenetics.

[65] Hsiao DJ (2009). Weight loss and body fat reduction under sibutramine therapy in obesity with the C825T polymorphism in the GNB3 gene. Pharmacogenet Genom.

[66] Grudell ABM (2008). A controlled pharmacogenetic trial of sibutramine on weight loss and body composition in obese or overweight adults. Gastroenterology.

[67] Hwang IC (2013). Effect of the G-protein beta 3 subunit 825T allele on the change of body adiposity in obese female. Diabetes Obes& Metab.

[68] Hattersley AT, Patel KA (2017). Precision diabetes: learning from monogenic diabetes. Diabetologia.

[69] Ahlqvist E (2018). Novel subgroups of adult-onset diabetes and their association with outcomes: a data-driven cluster analysis of six variables. Lancet Diabetes Endocrinol.

[70] Dennis JM (2019). Disease progression and treatment response in data-driven subgroups of type 2 diabetes compared with models based on simple clinical features: an analysis using clinical trial data. Lancet Diabetes Endocrinol.

[71] Dennis JM (2018). Evaluating associations between the benefits and risks of drug therapy in type 2 diabetes: a joint modeling approach. Clin Epidemiol.

[72] Dennis JM (2018). Precision Medicine in Type 2 Diabetes: Clinical Markers of Insulin Resistance Are Associated With Altered Short- and Long-term Glycemic Response to DPP-4 Inhibitor Therapy. Diabetes Care.

[73] MacLean PS (2018). The Accumulating Data to Optimally Predict Obesity Treatment (ADOPT) Core Measures Project: Rationale and Approach. Obesity.

[74] Alligier M (2020). OBEDIS Core Variables Project: European Expert Guidelines on a Minimal Core Set of Variables to Include in Randomized, Controlled Clinical Trials of Obesity Interventions. Obes Facts.

[75] Muller TD (2022). Anti-obesity drug discovery: advances and challenges. Nat Rev Drug Discov.

[76] Gomez G, Stanford FC (2018). US health policy and prescription drug coverage of FDA-approved medications for the treatment of obesity. Int J Obes.

[77] Atlas S, et al Medications for Obesity Management: Effectiveness and Value; Final Evidence Report. 2022, Inst Clin Econ Rev.

